# Music-Based Interventions in Paediatric and Adolescents Oncology Patients: A Systematic Review

**DOI:** 10.3390/children8020073

**Published:** 2021-01-21

**Authors:** Marta González-Martín-Moreno, Elisa María Garrido-Ardila, María Jiménez-Palomares, Gloria Gonzalez-Medina, Petronila Oliva-Ruiz, Juan Rodríguez-Mansilla

**Affiliations:** 1 Badajoz Association of Parents of Persons with Autism (Asociación de Padres de Personas con Autismo de Badajoz-APNABA), 06011 Badajoz, Spain; martaglzmm@gmail.com; 2 ADOLOR Research Group, Department of Medical-Surgical Therapy, Medicine Faculty, Extremadura University, 06011 Badajoz, Spain; mariajp@unex.es (M.J.-P.); jrodman@unex.es (J.R.-M.); 3 Nursing and Physiotherapy Department, Nursing and Physiotherapy Faculty, Cadiz University, Av. Ana de Viya, 52, 11009 Cádiz, Spain; gloriagonzalez.medina@uca.es (G.G.-M.); petronila.oliva@uca.es (P.O.-R.)

**Keywords:** music therapy, music-based interventions, child, adolescent, cancer, oncology, neoplasms

## Abstract

Background: The implications of cancer and its medical treatment are traumatic, highly stressful and have great psychosocial impact. Therefore, a comprehensive treatment is essential and music-based interventions can play an important role. The objective of this study is to summarise research that assesses the effects of music therapy in paediatric and adolescent patients with cancer during the process of the disease. Methods: A systematic review conducted following PRISMA’s statements. An electronic search of the literature was carried out in the following databases: PubMed, Cochrane, Dialnet, Scopus, IDICEs CSIC and Science Direct. Original studies that conducted music-based interventions with oncology patients between 0 to 18 years old were included. Results: 11 studies were finally included in the review. The sample consisted of two quasi-experimental studies, five randomised clinical controlled trials, one non-randomised controlled trial, one study that involved qualitative and quantitative analysis methods, one descriptive study and one observational study. Conclusions: Music-based interventions decrease anxiety, perceived pain and depression symptoms and improve state of mind, self-esteem and quality of life of paediatric and adolescent patients with cancer. Moreover, they decrease heart rate, respiratory rate and blood pressure and encourage patients to use adaptive coping strategies.

## 1. Introduction

Cancer is the second cause of death in children under 15 years old and the leading cause of death by disease in childhood. In spite of therapeutic advances, its incidence and prevalence have increased in recent years [1,2,3,4,5,6,7].

The experts and researchers highlight that being diagnosed with cancer is a very traumatic and highly stressful experience for children and adolescents. In addition, all the diagnostic tests, the treatments and the frequent hospital appointments have great psychosocial impact [8,9,10,11,12]. The worst experiences related to cancer are the pain related to the treatment and diagnostic tests, followed by depression, sleep disturbances, fatigue and anxiety [8,13].

The importance of and the need for pharmacological treatment such as radiotherapy and chemotherapy are unquestionable. However, it has become evident that these treatments can cause stress in children as their life is altered and they are constantly made aware of the disease [13,14]. This can lead to extreme negative behaviour such as screaming or poor collaboration which hinders the adherence to the treatment process [8,12,13,14].

The need for other complementary therapies to achieve a more effective and comprehensive treatment is being increasingly considered and studied [12,15]. These therapies include a wide range of approaches from psychological intervention, with cognitive-behavioural therapy, relaxation techniques or breathing exercises [10,12], to music therapy. Music has been used in different medical fields to meet the physiological, psychological and spiritual needs of patients [16]. According to the American Music Therapy Association, music therapy is defined as “a reflexive process wherein the therapist helps the client to optimize the client’s health, using various facets of music experience and the relationships formed through them as the impetus for change. As defined here, music therapy is the professional practice component of the discipline, which informs and is informed by theory and research” [17]. Such musical experiences may consist of listening to live, improvised or pre-recorded music, playing music on an instrument, improvising through voice or instruments, composing music, and using music combined with other modalities such as movement, images or art [16].

In addition, it is important to differentiate between the treatments implemented by a qualified music therapist (music therapy) and interventions that are categorised as “music medicine”. When the professional who carries out the intervention is a qualified music therapist, he or she tries to discover the child’s musical preferences, as well as to adapt to the child’s energy, needs and physical condition. [18]. In contrast, in a music medicine session, a health professional offers the patient passive listening to pre-recorded music [16].

The studies of music-based interventions that are available in the literature focus their intervention mainly on adults. Some authors have affirmed that music therapy significantly decreases anxiety levels and systolic blood pressure in oncology patients that undertake radiotherapy [19] or chemotherapy [20]. It has also been demonstrated that music therapy improves pain and anxiety in children that underwent lumbar puncture [11] and when symptoms are related to the hospitalisation process [21] or to the treatment sessions [22,23,24,25].

Based on this, the objective of this study was to analyse the effects of music-based interventions in paediatric and adolescent patients with cancer during the process of the disease (diagnosis, treatment and hospitalisation).

## 2. Materials and Methods

### 2.1. Study Design

This systematic review was carried out following the PRISMA statement [26]. The review protocol is available in PROSPERO (registration number: CRD42020204747). In order to identify relevant studies, the search was done in the following databases: PubMed, Cochrane, Dialnet, Scopus, InDICEs CSIC and Science Direct.

### 2.2. Search Strategy

The keywords used were: music therapy, child, adolescent, neoplasms, leukaemia, radiotherapy, chemotherapy, cancer and oncology. These keywords were introduced in Spanish when the databased required it. The Spanish terms used were: musicoterapia, neoplasias, cáncer, oncología, quimioterapia y radioterapia. The keywords were combined with the Boolean operators AND or OR.

### 2.3. Inclusion and Exclusion Criteria

The exclusion criteria were established following the PICO model (population, intervention, control and comparison and outcomes). The inclusion criteria were:

1. Type of participant: subjects within the age range 0 to 18 years old.
2. Type of intervention: music-based interventions as a complementary treatment.
3. Type of study: Randomised controlled trials, quasi-experimental studies, studies with experimental and control groups, or two experimental groups that had a sample of more than one participant and conducted more than one treatment session. The language of the studies was established to be English or Spanish. Due to the specificity of the subject and the lack of related scientific production, the date of publication in the searches was not limited.
4. Outcome measures: any outcome measure assessed with a standardised or validated assessment tool.

The exclusion criteria established were:

1. Systematic reviews, meta-analyses, studies with less than two treatment sessions or with less than four participants, study protocols, qualitative descriptions.
2. Absence of control group.
3. Participants over the age of 18 years.

### 2.4. Study Selection

**Figure 1:**
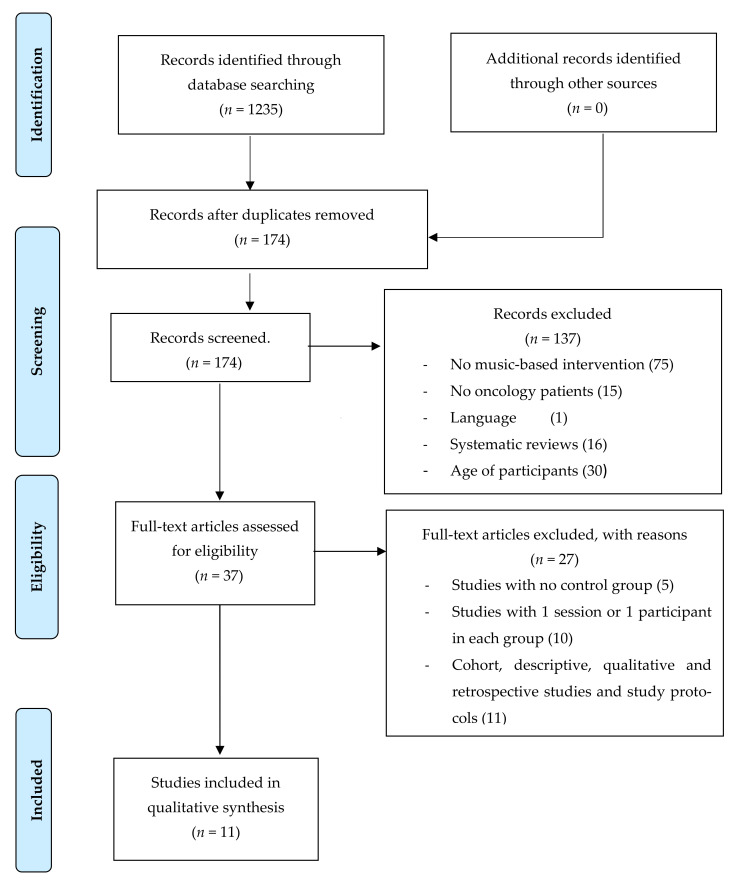
PRISMA flowchart.

A pre-selection of the papers was done considering that they were within the proposed subject of the study. This selection was carried out by reading the abstract of the studies and excluding those that did not meet the established criteria. The full text of the studies that did meet the inclusion criteria were revised, analysed and included in the systematic review. All potential full-text articles were retrieved and evaluated by the two reviewers independently. Although the level of agreement between the two reviewers was not specifically calculated, any disagreements on inclusion/exclusion of full-text articles were resolved by discussion (Figure 1).

The following data was obtained from the studies included in the review: characteristics of the sample, study design, description of the intervention and the control and experimental groups, outcome measures and results of the study. This data was compiled in a standard table. The reviewers who selected the articles also obtained the data and assessed the methodological quality of the studies. They did this independently and any disagreements were resolved by discussion.

### 2.5. Assessment of Methodological Quality

The analysis of the methodological quality of the studies was done using the PEDro (Physiotherapy Evidence Database) scale [27]. This consists of 11 items that can have a ‘yes’ (Y) or ‘no’ (N) as a reply. The total range of scores is from 0 to 10 according to a low to excellent methodological quality. The results obtained in the scale were considered as: High quality, if the score is over 5 (6–8: good, 9–10 excellent); Moderate quality, if the score is between 4 and 5 (fair quality study); Low quality, if the score is under 4 (poor quality study).

The first item is additional, related to the external validity, and is not used to calculate the score obtained. Therefore, the maximum score is 10. Items 2 to 9 aim to justify if the study has enough internal validity and items 10 and 11 analyse if the statistical information is appropriate to understand the results.

### 2.6. Risk of Bias Analysis

The risk of bias [28] was calculated for each included study, referring to the following types of bias: selection bias, performance bias, detection bias, attrition bias, reporting bias and other bias. The risk of bias and the quality of study were calculated by one reviewer only.

## 3. Results

The literature search was conducted in April 2015 and was updated in October 2020. A total of 1235 studies were obtained from the search in all databases. The PRISMA flow chart (Figure 1) shows the selection process of the studies. The records that were duplicated were excluded and 174 records were screened. Finally, 11 studies were included in the review.

The sample consisted of two quasi-experimental studies, five randomised clinical controlled trials, one non-randomised controlled trial, one study with a mix model design that involved qualitative and quantitative analysis methods, one exploratory and descriptive study and one observational study. All the papers were published between 1999 and 2019. Table 1 shows the main findings of this review.

### 3.1. Outcome Measures and Results

Regarding the sample size, the number of participants ranged from 8 to 240. Robb et al. [23,24] conducted the study with fewer participants and the study from Cabral-Gallo et al. [29] was the one that had the biggest sample.

In relation to the duration range of the music-based interventions, in the study by Nguyen et al. [11] the treatment consisted of the use of headphones with music 10 minutes before the lumbar puncture and during the procedure. In the case of Barry et al. [22], the intervention lasted from 10 to 90 minutes during the first radiation therapy session. As for the research of Robb et al. [23,24] the participants in the experimental groups received two music therapy sessions a week during three consecutive weeks. In Robb et al.’s [25] research conducted in 2008, the intervention consisted of a single 30-minute session. Cabral-Gallo et al. [29] carried out a group intervention with two hour sessions twice a week. In the study of Camprubí [30], the sessions of music-based intervention lasted 45-minutes and were performed while the patients were receiving chemotherapy or within 24 hours of its commencement. Uggla et al. (2018) [31] and Uggla et al. (2016) [32] performed 45-minute music therapy sessions twice a week over a period of four to six weeks. Giordano et al. [33] completed a study with a 15–20 minute music therapy session prior to a diagnostic procedure. Finally, Saghaeee-Shahriari et al. [34] conducted a total of 14 music therapy sessions with a duration of 90 minutes.

When analysing the use of music as an intervention tool, we could observe that eight of the selected articles [22,23,24,25,30,31,32,33] applied music therapy, as the professional who performed the intervention was a qualified music therapist. Three of the selected articles used music medicine, since the professional who performed it was not a qualified music therapist [29] or it was not specified [11,23,24,34].

Ten of the studies reviewed [11,22,23,24,25,29,30,31,32,33] applied the same type of intervention: music listening. Seven studies combined music listening with other techniques [22,23,24,25,30,31,33] such as the creation of a music therapy CD that includes preferred musical sounds [22], or composing [22]. Other authors compared music listening with the technique Active Music Engagement (AME) [24] or included music listening in a semi-structured session that involved singing, playing instruments or improvising [30,31,32,33].

The most highlighted outcome measure was anxiety as this was analysed in more studies [11,22,23,24,29,31,34]. Nguyen et al. [11] found a significant improvement in anxiety before and after lumbar puncture in the experimental group. The study conducted by Cabral-Gallo et al. [29] showed statistically significant differences in the physiological anxiety and hyper sensibility dimensions of the CMAS-R (Revised Children’s Manifest Anxiety Scale). Based on their results, they concluded that the carers of the experimental group perceived a significant improvement in the post-test compared with the pre-test in the following dimensions of the Hamilton Anxiety Scale (HAS): anxious humour, tension, fear, insomnia, mental functions, depressed humour, general somatic symptoms, somatic symptoms, cardiovascular symptoms, respiratory symptoms and gastrointestinal and autonomous nervous system symptoms. Giordano et al. [33] found a significant improvement in anxiety in the experimental group in comparison with the control group. Lastly, the results obtained by Saghaeee-Shahriari et al. [34] demonstrated that music therapy was effective in reducing sensibility to anxiety in adolescents with leukaemia.

In relation to quality of life, the findings of Uggla et al. [31] showed a significant difference in the Paediatric Quality of Life Inventory (PedsQL) in the experimental group after discharge in comparison with the control group. Other outcomes that showed improvements where the problems related to treatment concerns and anxiety.

Nguyen et al. [11] found significant pain relief during and after treatment. In the same way, the study of Uggla et al. [29] revealed that pain was reduced in the experimental group after the intervention. However, the differences were not statistically significant as compared with the control group.

Regarding state of mind, Camprubí [30] did not find any significant differences between the children from the experimental group and from the control group. In contrast, Uggla et al. [31] demonstrated that music therapy improved this outcome measure in comparison to the control group.

When analysing the changes in coping strategies, we can say that the study carried out by Barry et al. [22] did not show statistically significant differences between groups. It is interesting to highlight that they also found that social isolation was present only in the control group.

Regarding behaviour, the results from Robb et al. [25] revealed significant differences in active participation in the experimental group (AME) as compared to the control group (audition of music and audio books).

Vital signs improved significantly in the study of Nguyen et al. [11]. There were statistically significant differences in favour of the experimental group in heart rate and respiratory rate before lumbar puncture. After lumbar puncture there was also a significant difference in respiratory rate. Uggla et al. [32] found that the experimental group’s heart rate decreased over the course of the day, while the control group’s heart rate increased. The results showed that this difference between the groups was statistically significant. In addition, the evening heart rate of the experimental group was significantly lower than that of the control group. On the other hand, Camprubí [29] observed an improvement in immune system function in the pre-test and post- test comparison. Moreover, Camprubí [29] found an improvement of the immune system function in the pre-test and post- test comparison. Nevertheless, these differences were not statistically significant.

### 3.2. Methodological Quality of the Included Studies

The results of the methodological quality assessment can be seen in Table 2. It must be highlighted that eight studies [11,23,24,25,30,31,32,33] included in this review obtained a score ≥ 6 which indicates a good methodological quality while one study obtained a 5 [22] and 2 studies [27,32] obtained a score ≤ 4, indicating a mean quality. Random allocation was done in eight studies [11,22,23,24,25,30,31,32,34] and concealed allocation was performed in two studies [11,23]. The participants were blinded in only one of the papers reviewed [24] while the assessors were blinded in none of them.

### 3.3. Risk of Bias

The results of the risk of bias analysis can be observed in Table 3. It should be noted that eight of the selected articles [11,22,23,24,25,30,31,34] presented a low risk of selection bias, as they were randomized, although only three of them [11,22,25] also present allocation concealment. With respect to performance bias, only one [11] was low risk. Regarding detection bias, four of the articles included in the review [11,22,23,24,32] were low risk. In relation to dissertation bias, all of them [11,22,23,24,25,29,30,31,32,33,34] are low risk.

## 4. Discussion

This systematic review summarises the effects of music therapy in paediatric and adolescent oncology patients. Although there is a wide range of publications that analyse the effects of music therapy in oncology, there are very few that focus on children and adolescents. Besides, those that focus on these patients generally show qualitative results describing one or two cases [35,36].

We have observed that the music-based interventions that were not carried out by a qualified professional consisted of music listening by itself [11,29] or combined with the production of a video [23,24]. In contrast, the research conducted by music therapists had more developed interventions ranging from the creation of a music therapy CD and listening to it [22], the performance of live music sessions combined with dance, singing or games [30], the comparison of active musical participation with listening to music, and listening to an audio book, as in the study of Robb et al. [25]. This suggests that it is more convenient that the therapist in charge of the sessions is a qualified music therapist. This will ensure the correct development of the music therapy intervention, since they are the professionals who are specially, clinically and academically qualified. The quality of the interventions conducted by music therapists is probably based on the fact that they assess the specific needs of each patient and establish defined objectives and an individualised intervention plan. Moreover, as stated by Bradt et al. 2019 [37], qualified music therapists tend to involve patients more actively in the creation of music and to employ a systematic therapeutic process that includes assessment, treatment and evaluation.

In this sense, it should be noted that the absence of a qualified music therapist seems to be related to the simplicity of the treatment applied, since these other treatments are all based solely on music listening. An example of this is the study conducted by Cabral-Gallo et al. [29], who selected two pieces of classical music, two of folk music, one instrumental and one of medieval music. In contrast, Nguyen et al. [11] took into account the musical preferences of the participants, allowing then to select the music they wanted to listen to on the iPod. Saghaeee-Shahriari et al. [34] did not specify the music that the participants listened to.

In general, when the professional performing the intervention was a qualified music therapist the participants were more involved in the selection of the music and more motivated to get actively involved in the treatment. In Robb et al.’s research [23,24] the interventions were specifically designed to provide patients with the opportunity to make independent choices and decisions, express feelings related to identity and/or hospitalisation, provide multi-sensory stimulation and participate in a goal-oriented intervention. In the study carried out by Robb et al. in 2008 [25] children were given numerous opportunities to choose materials for the active musical experience sessions. As the treatment and the sessions were guided by a qualified music therapist who focused on supporting the children’s decisions, it also promoted their autonomy. Camprubí [30] performed music therapy sessions that were adapted to the musical preferences of the participants. This was achieved by allowing the participants to either select the type of song or the type of instruments for the sessions. In the study of Uggla et al. (2016) [32], the music therapist adapted the music they listened to according to the results of an assessment of the participants. Moreover, Uggla et al. (2018) [31] highlighted in their study the importance of the music therapist’s role, who was responsible for discovering the child’s musical preferences as well as adapting to the child’s energy, needs and physical condition. Finally, Barry et al. [22] allowed the children who participated in their study to choose and create the sounds they liked best for the development of the CD.

As explained by Stegemann et al. [38], music therapy and music-based interventions are particularly effective in childhood and adolescence in improving mood, regulation, communication, social skills and quality of life and, in addition, musical interventions carried out in medical settings (music medicine) manage to relief pain, anxiety and stress. Therefore, due to the variety of symptoms that music-based interventions can affect, this discussion is structured by the outcome measures analysed by the studies included in the review.

### 4.1. Music-Based Interventions and Anxiety

After the revision of the bibliography, the evidence suggests that there is a relation between music therapy and anxiety reduction. Music therapy encourages the expression of feelings and thoughts which leads to a decrease in anxiety symptoms [39]. Two studies [11,24] found significant improvements in children and adolescent anxiety after receiving music therapy based on musical audition. Nguyen et al. [11] studied anxiety levels before and after lumbar puncture and related the low scores obtained by the experimental group to the fact that music helps to control unpleasant situations such as invasive procedures performed for the treatment of cancer. These results coincide with those obtained in other research conducted with adults [40,41]. They are also consistent with a previous Cochrane review [39] which included 30 papers that analysed the physical and emotional effects of music therapy in cancer patients. The results indicated a significant improvement in anxiety. In addition, Cabral-Gallo et al. [29] found significant positive changes in two dimensions of anxiety: physiological anxiety and hyper-sensibility. The authors considered that these changes could be related to the fact that music can decrease the sensation of a hostile environment that can be associated with hospitalisation.

Moreover, this seems to be in line with various authors, such as Wang [42], whose research showed a clear link between music and emotions, since both harmony and tempo can affect the emotional meaning of a piece of music. For example, major chords are typically experienced as happy and minor ones as sad and slower music seems less happy than faster rhythms. All this can cause music to arouse feelings and associated physiological responses. Furthermore, in the research conducted by Nikjeh et al. [43], it was found that formally trained musicians showed, in comparison to non-musicians, more efficient neural tone detection, superior auditory sensory memory traces for acoustic characteristics, and greater sensitivity to acoustics. This suggests that music training can influence central auditory function and modulate the auditory neural system.

### 4.2. Music-Based Interventions and Quality of Life

One of the studies that were included in this systematic review [31] analysed the effects of music therapy on the quality of life of paediatric and adolescent oncology patients. They observed, that after the music therapy intervention, children and adolescents experienced less problems related to treatment concerns and anxiety [29]. These results can be related to those obtained in a Cochrane review [37], which explained how the interventions do not always lead to significant changes in quality of life, as this outcome is associated with many other aspects such as physical symptoms, pain and psychological symptoms (for example, anxiety or state of mind).

### 4.3. Music-Based Interventions and Pain

Only two of the reviewed studies [11,31] analysed the relation between pain and music-based interventions. These authors found statistically significant improvements in this outcome measure during and after lumbar puncture [11] and after hematopoietic stem cell transplant [31]. They [11,31] concluded that music can distract the person and, in particular, audition of familiar songs reminds the person of previous pleasant situations which decreases perceived pain. These results coincide with those obtained in other studies conducted with adults [39,40]. Zengin et al. [39] observed the significant benefits of music therapy in adults who had pain related to a catheter placement. Shabanloei et al. [40] also found that music therapy decreased pain in patients during bone marrow aspiration and biopsies.

These results seem to be related to the research conducted by Hole et al. [44], Garza-Villareal et al. [45] and Lee et al. [46]. Hole et al. [44] concluded that music could reduce preoperative pain and anxiety. Lee et al. [46] affirmed, after reviewing 97 trials, that musical interventions appear to have beneficial effects on pain intensity and emotional distress due to pain. Garza-Villareal et al. [45] also highlighted that when music is chosen by oneself, it appears to have a greater analgesic effect than music chosen by the researchers.

In the study carried out by Dobek et al. [47], the authors went further into this aspect of music’s effect and investigated neural mechanisms during the application of a painful stimulus while participants were listening to their favourite music. After mapping the neuronal responses in the brain, the brain stem and the spinal cord, they found that music seemed to affect spinal nociceptive responses. This suggests that listening to music can effectively block nociceptive processing in the dorsal shaft of the spinal cord compared to a condition without music. In this regard, Pando-Naude et al. [48], who investigated the effects of music on patients with fibromyalgia, highlighted that patients who listen to music showed a decrease of resting state functional connectivity (rs-FC) of the pain matrix. They also observed changes in the anterior cingulate cortex, which is involved in processing the affective and unpleasant aspects of pain. This indicates that music has the ability to reduce the connectivity of the anterior cingulate cortex with the sensory areas, thus reducing the perception of pain. In addition, the study describes the default mode network, whose set of areas is most active when the person is in a state of mental distraction. This suggest that music produces pain relief which could be related to analgesic effect. In relation to the dorsolateral prefrontal cortex, Pando-Naude et al. [48] described that listening to music modulates the perception of pain through cognitive control, which is also correlated with analgesia. Finally, they also emphasised that music influences the limbic areas which are involved in emotion, attention, learning, memory and motivation. These positive effects seem to be consistent with the use of a familiar, pleasant and emotionally positive piece of music.

Moreover, the scientific literature evidences that music stimulates the release of endorphins [49]. Endorphins are neurotransmitters that mask pain, which could be the reason why music therapy reduces pain and its related symptoms. Further research should be conducted in order to analyse if music therapy could be beneficial in decreasing analgesics intake and therefore could reduce the secondary effects of the medication and the cost of the treatment for cancer. Actually, Fernández [50] published a study protocol whose objective is to compare the effectiveness of music therapy and local anaesthetics for pain relief during lumbar puncture of children between 5 and 12 years old.

### 4.4. Music-Based Interventions and Depression

Regarding depression, one study [33] analysed the effects of music therapy on this disorder. The authors completed a qualitative description of the changes experienced in relation to the depressive symptoms during the different phases of treatment with invasive procedures. After their analysis, they concluded that music therapy caused an improvement in symptoms. Only one study [23] analysed with statistical methods the effects of music therapy on depression and found improvements in the experimental group. The results could be justified, as music seems to improve communication and environmental interaction. This can avoid the appearance of depressive behaviours and loss of social contact related to the difficulties that a patient can find in his or her adaptation to the environment [47].

### 4.5. Music and State of Mind

State of mind was analysed by Camprubí [30], although this author did not find statistically significant differences after the music therapy intervention. In contrast, the results obtained by Uggla et al. [31] were positive in relation to the improvements in state of mind. The improvement seems obvious and could be due to improvement in self-esteem, and reduction of pain and depressive symptoms.

### 4.6. Music-Based Interventions, Coping Strategies and Behaviour

Of two studies that found benefits in behaviour and coping strategies [22,25], only one [25] observed significant changes after musical audition and AME. These changes could be related to the improvement of state of mind that facilitates the use of coping strategies and at the same time could lead to better behaviour. Barry et al. [22] described the percentage of participants that used each type of coping strategy but only found statistically significant differences in relation to social isolation (only present in the control group). This could be explained by the fact that music therapy facilitates communication and interaction of the patient with the therapist and the family. This interaction is key to avoid social isolation, very common in cancer patients.

### 4.7. Music-Based Interventions and Vital Signs

The positive results obtained by Nguyen et al. [11] and Uggla et al. [32] in relation to vital signs coincide with those published in a Cochrane review conducted by Bradt et al. [37]. Music therapy improved heart rate, respiratory rate and blood pressure which resulted in the relaxation of the patient.

### 4.8. Study Limitations

The fact that there are very few experimental studies that analyse the effects of music therapy in children and adolescents with cancer can be considered a limitation of this review. The established inclusion criteria could have influenced the number of studies found and finally included in the review. In particular, the language of publication, including only articles in English or Spanish, may have led to the exclusion of relevant studies. However, even though the rest of the criteria were established to ensure homogeneity of the sample as much as possible, the results of the review showed that the studies were heterogeneous and dealt with different age groups. In addition, the studies analysed were heterogeneous in relation to the interventions, outcome measures and measurement tools. This made the comparison of the results, the studies and the effectiveness of the interventions very difficult.

Regarding the methodological quality, the studies included in this review obtained a score between 4 and 8 on the PEDro scale. According to the PEDro interpretation guidelines, if the studies had a score of at least 5 out of 10 they were considered to be of an acceptable quality. Studies that obtained scores of around 4 did not include the blinding of all patients, therapists and assessors. Due to the nature of music therapy interventions, it is very difficult to have triple blinding as no placebo can be used and the treatment provided is clear for the therapists. However, the assessor could have been blinded in most of the studies, except one [21] in which the therapist was also the assessor.

The results of the present review can have important implications in clinical practice. Our data shows that music-based interventions have many positive effects on paediatric and adolescent oncology patients such as improvement in quality of life [30]; decrease in concerns and anxiety related to the treatment; improvement of behaviour [24]; improvement in mood [30] and coping strategies [21], particularly in social isolation; decrease in anxiety [21]; and improvements in immune function [29] and evening and morning heart rate and saturation [31]. This suggests that these treatment approaches could be used as a complement to medical treatment. We also consider that these findings should be interpreted with caution as the results obtained in the studies analysed are very heterogeneous. However, we have observed that the interventions carried out by a music therapist [21,22,23,24,29,30,31,32] obtained benefits more effectively than the interventions that were not conducted by a music therapist [11,28,33]. Despite this, there are other music-based interventions such as music listening that have shown improvements in relation to anxiety [11,28,33], pain, and heart and respiratory rate [11]. These interventions are being increasingly used by health professionals, have low cost and can be performed safely and effectively as a complement to the oncological treatment.

Further research on this technique and its effects in paediatric and adolescent oncology patients is required. We suggest that future studies must have a greater sample size, homogeneous criteria such as cancer type and stage, and appropriate methodological quality. This would allow the appropriate analysis of the effects of music therapy, the development of treatment protocols and the extrapolation of results to other populations in order to improve the quality of life of the patients.

## 5. Conclusions

Based on the results of the studies analysed, music-based interventions improve anxiety and pain in paediatric and adolescent oncology patients during diagnostic procedures and during hospitalization.

Music-based interventions improve state of mind and self-esteem, decrease depressive symptoms during the cancer treatment, stimulate adaptive coping strategies by decreasing social isolation during radiotherapy and hospitalization, and improve quality of life.

Music-based interventions have beneficial effects on vital signs, decreasing heart rate, respiratory rate and blood pressure before and after lumbar puncture.

Listening is the technique most frequently used in the studies. However, when a qualified music therapist was involved in the intervention, more complex techniques such as active music engagement were used.

However, given the heterogeneity of the studies, it is complex to extrapolate results in this review.

## Figures and Tables

**Table 1:** Main characteristics of the studies.

Author	Age, Mean (SD), Median	Sample Size	Type of Intervention	Outcome Measures/ Assessment Tools	Results
Nguyen et al. [11]	EG = 7–12, 8.8 (1.59) CG = 7–12, 9.4 (1.93)	N = 49 (9 looses) EG= 20 CG = 20	EG = music on the iPod with headphones CG = headphones with no music	Vital signs. NRS STAIC	EG: pain relief (*p* < 0.001) during (*p* < 0.003) and after the interventions EG: reduction of the anxiety (*p* < 0.001) before and after the LP EG: Changes in HR (*p* = 0.012) and RR (*p* = 0.009) before LP an in HR after LP (*p* = 0.003)
Barry et al. [22]	EG = 6–13, median: 8 CG = 6–13, median: 8	N = 12 (1 loss) EG = 5 CG = 6	EG: creation of a MT CD CG: no MT treatment	Coping strategies Kidcope questionnaire	No significant differences regarding anxiety during RT. No significant differences regarding coping strategies in both groups. Significant differences in relation to social isolation (*p* = 0.076) which was only present in the CG.
Robb et al. [23]	EG = 9–17 CG = 9–17	N = 8 (1 loss, 1 excluded) EG = 3 CG = 3	EG: 3 active sessions of composition, discussion and songs recording + three passive sessions to avoid fatigue and secondary effects CG: The person participated in one of the following activities of his/her choice: (a) table game, (b) cards game, (c) videogame.	STAIC CDI	All participants in the EG showed lower anxiety levels. The results in the CG were very variable.
Robb et al. [24]	EG = 9–17 CG = 9–17	N = 8 (1 loss, 1 excluded) EG = 3 CG = 3	EG: three active sessions of composition, discussion and songs recording + three passive sessions to avoid fatigue and secondary effects CG: The person participated in one of the following activities of his/her choice: (a) table game, (b) cards game, (c) videogame.	STAIC CDI Likert scale	Four independent readers identifying themes in patient-generated songs, predominant categories for P1’s lyrics included themes related to control or independent coping (19%), hope (14%) and family support (11%); P2′s lyrics included positive physical status (58%), negative physical status (38%), positive mental status (33%), and professional/staff support (19%); P3′s lyrics included family support (75%) and appreciation (38%). Through a Likert scale they evaluated how the condition affected their stay in hospital (5 = very useful, 3 = neutral, 1 = harmful). The results showed that music helped them to use the time for fun (M = 5), and with an average score of 4.5 that “encouraged me to make choices”, “helped me feel good about myself”, “improved my mood”, and “helped me express my thoughts and feelings”.
Robb et al. [25]	EG = 4–7 CG = 4–7 CG = 4–7	N = 83 (1 excluded) EG AME = 27 CG ML 28 CG AB = 28	EG AME: five-part session (greeting, playing and instrument, movement while listening to a song, songs and stories, song to close the session) CG ML: listening to a CD of music CG AB: listening to an audiobook for 10–15 min	Behavioural coding: - Facial expression, - Active participation - Behaviour - Initiation: verbal and gestural	Significant differences on facial expression between the EG and both CG (*p* < 0.0001). Significant differences between both CG (*p* < 0.0413). Significant differences in active participation between the EG and both CG (*p* < 0.0001 both), no significant differences between both CG (*p* = 0.9527). The CG ML showed the best scores in initiation, followed by EG and CG AB. The difference between the CG ML and the CG AB was significant (*p* = 0.0019). However, the difference between the EG and the CG ML was not (*p* = 0.5552).
Cabral-Gallo et al. [29]	EG = 6–18 CG = 6–18	N = 240 (112 patients, 128 carers) EG = 56 CG = 56	EG: music listening CG: no MT treatment	Patients: C-MAS-R Carers: HAS	Mean value of anxiety decreased in the pre-test post-test comparison in the EG (12.71 y 11.95) and the CG (13.89 y 13.21). Both effect sizes were small (0.20 y 0.19). Significant differences in the EG in the physiological anxiety (*p* = 0.004) and Hyper sensitivity (*p* = 0.028) dimensions in girls. In the CG there were significant differences in physiological anxiety (0.043). Carers: Statistically significant changes in the EG (*p* < 0.05) in 12 of the 13 dimensions. Only two dimensions showed significant changes in the CG.
Camprubí [30]	EG = 5–16 CG = 5–16	N = 30 EG = 15 CG = 15	EG: 45 minutes MT session (live music, simple melody expressive songs, dancing, free drawing, musical games with any family member) CG: 45 minutes of a leisure activity adapted to the age of the patient (avoiding music)	Immunoglobulin A in saliva Likert scale	EG: increase of 7 mg/L compared to the pre-test, although the changes were not significant. No significant differences (*p* > 0.05) in state of mind in the EG (pre-test post-test difference of 0.6) and the CG (increase of 0.4 between the pre-test and the post-test). The difference between the EG and the CG was not significant (*p* > 0.05).
Uggla et al. [31]	EG = 7.1 (0.5–17) CG = 6.2 (0.2–16)	N = 71 (35 excluded, 7 losses) EG = 14 CG = 15	EG: 45 minutes’ sessions, twice a week during hospitalisation (4–6 weeks). The children were invited to sing, play musical instruments and listen to music. CG: No MT treatment.	PedsQL Performance scale in the game of Lansky	After discharge, the EG showed a significant difference (*p* = 0.0079) in the physical function domain of the PedsQL as compared with the CG. In addition, the EG had less treatment concerns and anxiety (*p* = 0.41 y *p* = 0.17; respectively). The state of mind in the EG improved significantly after music therapy (*p* = 0.000) in comparison with the CG. Pain decreased in the EG after the intervention but the changes were not statistically significant as compared to the CG.
Uggla et al. [32]	EG = 6 (0.9–16) CG = 6 (0.2–14)	N = 40 (16 declined, 3 losses) EG = 12 CG = 9	EG: 45 minutes’ sessions, twice a week during hospitalisation (4–6 weeks), according to the Nordoff-Robbins Creative Music Therapy and Juliette Alvin’s Free Improvisation Therapy. The child is active and is invited to sing, play various musical instruments and listen to music with the therapist CG: no MT treatment.	Vital signs. Performance scale in the game of Lansky	Significant differences between the evening and morning heart rate of the EG with respect to the GC (*p* <0.001) were found. Significant difference in saturation, with lower EG scores, with respect to CG (*p* = 0.06) were observed. However, the night the scores are similar. No significant differences in blood pressure were observed between the groups (*p* = 0.46)
Giordano et al. [33]	EG = 2–13 CG = 2–13	N = 48 EG = 29 CG = 19	EG: one MT session of 15 to 20 minutes (playing musical instruments, improvisation, singing, musical creation, selection and play of music playlists). CG: entertainment with leisure activities	m-YPAS Likert scale	The EG had significant less anxiety levels after the intervention in comparison to the CG. 66.7% of the interviewed answered“very much“, 30.3% "a lot“ and 3% “sufficiently“ in the question related to the ability to distract the patients.
Saghaeee-Shahriari et al. [34]	EG = adolescents, ages not specified. CG = adolescents, ages not specified.	N = 30 EG = 15 CG = 15	14 sessions of music therapy lasting 90 minutes	ASI General self-efficiency scale	Significant difference in the EG in anxiety sensitivity (*p* < 0.001) and self-efficiency (*p* < 0.001) in comparison to the CG.

SD: Standard deviation. EG: Experimental Group. CG: Control Group. LP: lumbar puncture. MT: Music Therapy. CD: Compact disc. P: Participant. NRS: Numeric Rating Scale for pain assessment. HR: Heart rate. RR: Respiratory rate. RT: Radiotherapy. STAIC: State-Trait Anxiety Inventory for Children. CDI: Children depression inventory. P: Participant. AME: Active music engagement. ML: music listening. AB: Audiobook. M: Media. C-MAS-R: Children’s Manifest Anxiety scale revised. HAS: Hamilton Anxiety Scale. PedsQL: Paediatric Quality of Life Inventory. m-YPAS: modified Yale Preoperative Anxiety Scale. CES-DC: Centre for Epidemiological Studies Depression Scale for Children and Adolescents (Chinese version). ASI: Anxiety Sensitivity Index.

**Table 2:** Physiotherapy Evidence Database (PEDro) scale.

Study	Criteria											Score
	1	2	3	4	5	6	7	8	9	10	11	
Nguyen et al. [11]	S	S	S	S	N	N	N	S	S	S	S	7
Barry et al. [21]	S	S	N	S	N	N	N	S	S	S	S	5
Rob et al. [22]	S	S	S	S	N	N	N	S	S	N	S	6
Robb et al. [23]	S	S	S	S	N	N	N	S	S	N	S	6
Robb et al. [24]	S	S	N	S	S	N	N	S	S	S	S	8
Cabral-Gallo et al. [28]	S	N	N	N	N	N	N	S	S	S	S	4
Camprubí [29]	S	S	N	S	N	N	N	S	S	S	N	6
Uggla et al. [30]	S	S	N	S	N	N	N	S	N	S	S	6
Uggla et al. [31]	S	S	N	S	N	N	N	S	N	S	S	6
Giordano et al. [32]	S	N	N	N	N	N	S	S	S	S	S	6
Saghaeee-Shahriari et al. [33]	N	S	N	N	N	N	N	S	S	S	N	4

N: Did not met the criteria; S: Met the criteria. 1. Eligibility criteria were specified; 2. Random allocation; 3. Concealed allocation; 4. Similar groups at baseline; 5. Blinding of all subjects; 6. Blinding of all therapists; 7. Blinding of all assessors; 8. Follow up of more than 85% of the subjects; 9.Intention to treat analysis; 10. Between-group statistical comparisons; 11. Point measures and measures of variability for at least one key outcome are given.

**Table 3:** Risk of Bias.

Study	Criteria						
	1	2	3	4	5	6	7
Nguyen et al. [11]	+	+	+	+	+	+	+
Barry et al. [21]	+	+	N/A	?+	+	+	+
Rob et al. [22]	+	?	N/A	+	+	+	+
Robb et al. [23]	+	?	N/A	+	+	+	+
Robb et al. [24]	+	+	N/A	?	+	+	+
Cabral-Gallo et al. [28]	-	-	N/A	-	+	+	+
Camprubí [29]	+	-	N/A	-	+	+	?+
Uggla et al. [30]	+	?	N/A	?	+	+	+
Uggla et al. [31]	?	?	N/A	+	+	+	+
Giordano et al. [32]	-	-	N/A	?	+	+	+
Saghaeee-Shahriari et al. [33]	+	?	N/A	?	+	+	+

+ = “Low risk” of bias; - = “High risk” of bias; ? = “Unclear risk” of bias; N/A = Not Applicable.1 = Random sequence generation (selection bias). 2 = Allocation concealment (selection bias). 3 = Blinding of participants and personnel (performance bias). 4 = Blinding of outcome assessment (detection bias).5 = Incomplete outcome data (attrition bias). 6 = Selective reporting (reporting bias). 7 = Other bias.
